# Supplementary material for: Pathophysiological Significance of Neutrophilic Transfer RNA-Derived Small RNAs in Asymptomatic Moyamoya Disease
Source: Cells. 2021 May 1;10(5):1086. doi: 10.3390/cells10051086 (PMC8147334; doi:10.3390/cells10051086)

2D Structure | **Local AU** | **Position**

```
1499                        8mer-1a      1529
 5'-gttACTGTTTTCCACTTAGCTCTGTGTGGGa-3' utr
      |  |  :|   ||  |  |||   |::|||||||
 3'-uccUUAGGAUUGGCG-AUC-UGGUACACCCu-5' tRF
              1615  1413      7 6 5 4 3 2
              3' Pairing        Seed
```

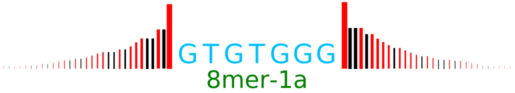

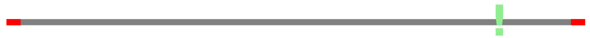

Supplement: Supplementary file 1 [file cells-10-01086-s001.zip › cells-1149161 3rd supplementary materials/Data Supplements/Data Supplement V.zip!/Data supplement 6-mRNAs targets of tsRNAs/tsRNA-Glu-TTC-010/Diagrams/tRF-Glu-TTC-010_vs_ENST00000228872(CDKN1B).pdf]
